# A novel composite inflammatory–metabolic index for long-term prognosis after TAVR: the modifying role of left ventricular ejection fraction

**DOI:** 10.3389/fcvm.2026.1912257

**Published:** 2026-08-25

**Authors:** Haitham Abu Khadija, Nizar Abu Hamdeh, Duha Najajra, Kareem Ibraheem, Jebrin Alkrinawi, Osama Hroub, Yahya Z. Fraitekh, Mohammad Masu'd, Abdalaziz Darwish, Mohammad Alnees

**Affiliations:** 1 Heart Center, Kaplan Medical Center, Rehovot, Affiliated with the Hebrew University, Jerusalem, Israel; 2 Department of Internal Medicine B, Kaplan Medical Center and Faculty of Medicine, Hebrew University of Jerusalem, Jerusalem, Israel; 3 Palestinian Clinical Research Center, Bethlehem, Palestine; 4 Postgraduate Medical Education, Global Clinical Scholar Research Training Program, Harvard Medical School, Boston, MA, United States

**Keywords:** composite biomarker, hemodynamic valve deterioration, left ventricular ejection fraction, mortality, systemic immune-inflammation index, transcatheter aortic valve replacement, TyG index

## Abstract

**Background:**

Metabolic dysfunction and systemic inflammation are increasingly recognized as important determinants of outcomes after transcatheter aortic valve replacement (TAVR). However, whether their prognostic impact differs according to baseline left ventricular systolic function remains unclear.

**Objectives:**

To evaluate the association between the TyG–SII composite index (TSI), an inflammatory–metabolic biomarker, and long-term outcomes after TAVR and to determine whether baseline left ventricular ejection fraction (LVEF) modifies this association.

**Methods:**

We conducted a retrospective cohort study of patients who underwent TAVR between December 2015 and March 2025. Patients were stratified according to baseline LVEF (≤40% vs. >40%). To reduce confounding, a 1:4 exact matched cohort was constructed according to sex, age category, calendar period of TAVR, and valve type (*n* = 646). TSI was calculated from pre-procedural laboratory values and analyzed as a continuous standardized variable (per 1-SD increase). Long-term all-cause mortality was assessed using Cox proportional hazards models stratified by LVEF. A separate 1:4 time-dependent matched cohort (*n* = 224) was used to evaluate the association between LVEF and long-term hemodynamic valve deterioration (HVD).

**Results:**

During follow-up, 187 deaths occurred: 51 among patients with LVEF ≤ 40% (33.3%) and 136 among those with LVEF > 40% (27.6%). Kaplan–Meier analysis showed no significant difference in overall survival between LVEF groups (log-rank *p* = 0.225). In multivariable analyses, each 1-SD increase in standardized TSI was independently associated with higher mortality among patients with LVEF ≤ 40% (adjusted HR, 1.38; 95% CI, 1.10–1.73; *P* = 0.005), but not among those with LVEF > 40% (adjusted HR, 1.02; 95% CI, 0.84–1.24; *P* = 0.818). Patients with reduced LVEF also exhibited a higher long-term risk of HVD compared with matched controls (adjusted HR, 1.71, 95% CI, 1.00–2.91, *p* = 0.048).

**Conclusions:**

In patients undergoing TAVR, greater inflammatory–metabolic burden, as reflected by TSI, was independently associated with long-term mortality among those with reduced LVEF. Reduced LVEF was also associated with a higher long-term risk of HVD; however, this exploratory finding requires confirmation in larger prospective studies.

## 1 Introduction

The clinical landscape of TAVR has evolved substantially over the past decade, with advances in device design, patient selection, and procedural techniques contributing to improved short- and mid-term outcomes across surgical risk groups (1, 2). Within this context, LVEF remains a key clinical marker for risk stratification in patients with aortic stenosis undergoing TAVR, because of its established association with mortality and symptomatic status. However, its prognostic value remains incomplete, as baseline LVEF alone may not fully capture the effects of systemic comorbidities, myocardial vulnerability, and the complex inflammatory–metabolic milieu that influence long-term outcomes after TAVR (3, 4).

A growing body of evidence supports the role of systemic inflammation and metabolic dysfunction as key determinants of adverse cardiovascular outcomes, including progression of atherosclerosis, heart failure, and mortality across cardiometabolic conditions. The triglyceride–glucose (TyG) index, a surrogate marker of insulin resistance derived from fasting triglycerides and glucose levels, has demonstrated consistent associations with cardiovascular risk and overall mortality in large epidemiologic cohorts, reflecting the importance of insulin resistance in cardiovascular pathogenesis (5, 6). Similarly, the systemic immune-inflammation index (SII), calculated from neutrophil, lymphocyte, and platelet counts, has emerged as a prognostic biomarker linked to all-cause and cardiovascular mortality in large population-based studies, emphasizing the contribution of immune dysregulation on clinical outcomes (7). Although TyG and SII each have independent prognostic value, they have largely been evaluated separately, focusing on isolated metabolic or inflammatory pathways rather than their combined effects. Studies integrating TyG with markers of systemic inflammation, including composite indices like TyG-SII, suggest that integrating metabolic and inflammatory burden may better capture their synergistic association with adverse clinical outcomes than either index alone (8, 9).

Despite growing recognition of the prognostic relevance of systemic inflammation and metabolic dysfunction in patients with severe aortic stenosis and those undergoing TAVR, existing evidence remains largely fragmented, focusing on isolated biomarkers rather than integrated biological pathways. Prior studies have evaluated inflammatory indices such as C-reactive protein, neutrophil-to-lymphocyte ratio, or systemic immune-inflammation index, as well as metabolic markers including diabetes status or insulin resistance surrogates, with variable and sometimes inconsistent associations with long-term outcomes after TAVR (10–12). Recent TAVR literature has increasingly emphasized the value of composite biomarkers that integrate different biological domains rather than relying on isolated laboratory parameters. For example, Buonpane et al. demonstrated that the platelet-to-lymphocyte/albumin ratio, a combined inflammatory–nutritional marker, was independently associated with one-year all-cause mortality after TAVR and outperformed the prognostic value of single parameters (13). However, evidence specifically evaluating an integrated inflammatory–metabolic composite based on TyG and SII in the TAVR population remains limited. Moreover, it remains uncertain whether the prognostic impact of such biological stressors differs according to baseline left ventricular systolic function, a key determinant of myocardial reserve and post-procedural recovery. This represents an important gap in knowledge, particularly given the heterogeneous long-term outcomes observed after TAVR despite similar procedural success.

Accordingly, the present study aimed to investigate the prognostic impact of the TyG–SII composite index in patients undergoing TAVR, with a particular focus on the modifying role of left ventricular systolic function. Specifically, we examined the association between inflammatory–metabolic burden and long-term all-cause mortality, and explored whether reduced left ventricular ejection fraction is associated with an increased risk of hemodynamic valve deterioration during follow-up.

## 2 Methods

### 2.1 Study design and population

This retrospective cohort study included patients undergoing TAVR at our institution between December 2015 and March 2025. All patients with severe symptomatic aortic stenosis who received a bioprosthetic transcatheter valve during this period were considered for inclusion. Baseline LVEF measured before TAVR was used to define the exposure groups: reduced LVEF (≤40%) and LVEF > 40%.

**Figure 1:**
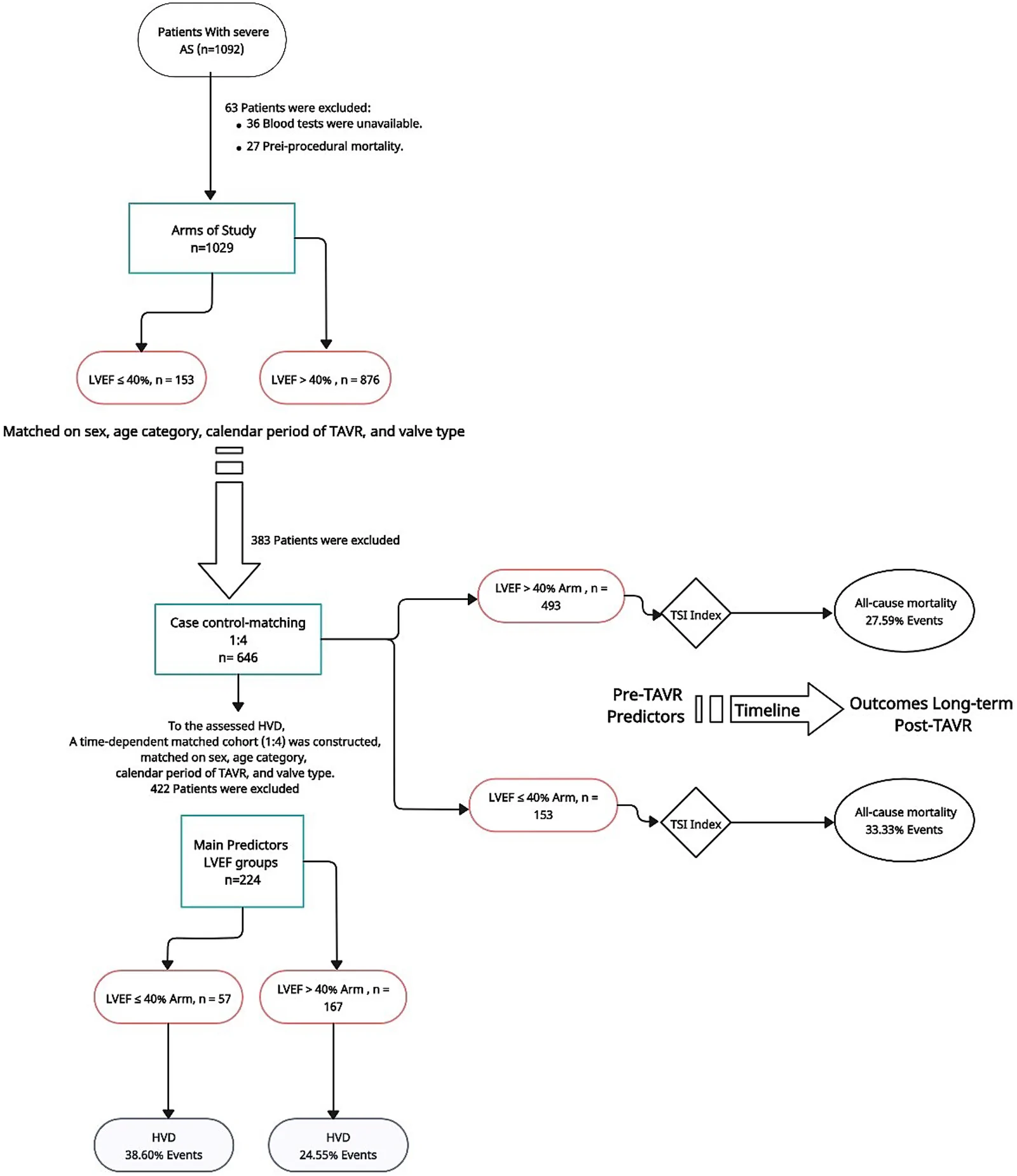
Study flow diagram.

Figure 1 illustrates the study population selection, matching strategy, and analytical framework. Two separate matched analytic cohorts were constructed for the primary and secondary study objectives. For the primary analysis of long-term all-cause mortality, a 1:4 exact matched cohort was created by matching patients with LVEF ≤ 40% to patients with LVEF > 40% according to sex, age category, calendar period of TAVR, and valve type. This cohort was used to evaluate the association between standardized TSI and long-term mortality within each LVEF stratum. For the secondary analysis of HVD, a separate 1:4 time-dependent matched cohort was constructed among patients with adequate longitudinal echocardiographic follow-up, allowing assessment of long-term valve hemodynamics. This cohort was used specifically to evaluate the association between baseline LVEF and subsequent HVD.

A total of 1,092 patients with severe aortic stenosis were initially identified. Sixty-three patients were excluded because of unavailable blood tests (*n* = 36) or pre-procedural mortality (*n* = 27), leaving 1,029 patients eligible for analysis. Patients were stratified according to LVEF into the LVEF ≤ 40% group (*n* = 153) and the LVEF > 40% group (*n* = 876). For the primary mortality analysis, exact matching resulted in the exclusion of 383 patients and the construction of a 1:4 matched cohort of 646 patients. The study was approved by the institutional review board (Approval No. 0045-25), which waived the requirement for informed consent because of the retrospective design.

The cohort included in the present study partially, but not completely, overlaps with an institutional TAVR cohort previously reported by our group in a study evaluating the association between the triglyceride–glucose index and mortality after TAVR (14). The previous study included 821 patients treated between December 2010 and March 2024, whereas the present study included patients treated between December 2015 and March 2025. Accordingly, overlap was limited to patients treated during the shared calendar period who met the distinct eligibility criteria of both studies. The cohorts were not identical because the previous study included patients treated during the earlier institutional TAVR era from 2010 to 2015, whereas the present study included additional patients treated after March 2024 and applied different exclusion criteria and cohort-construction methods. The previous publication evaluated TyG alone in relation to 30-day, 1-year, and 3-year all-cause mortality and did not evaluate SII, construct or analyze the TyG–SII composite index, assess its interaction with baseline LVEF, or examine hemodynamic valve deterioration. All analyses involving SII, TSI, the TSI × LVEF interaction, and HVD reported in the present study are novel and have not been previously reported.

### 2.2 Exposure definition: TyG–SII composite index (TSI)

Pre-procedural fasting blood samples, obtained as part of the routine pre-TAVR evaluation, were used to calculate the TyG–SII composite index (TSI), which reflects the combined metabolic and inflammatory burden. The triglyceride–glucose (TyG) index was computed as ln[fasting triglycerides × fasting glucose/2], and the systemic immune-inflammation index (SII) was defined as platelet count × neutrophil count/lymphocyte count. These component indices have been validated in prior studies as prognostic biomarkers of cardiovascular risk (14, 15). We derived TSI by multiplying the TyG and SII indices to generate a composite measure reflecting the concurrent burden of metabolic dysfunction and systemic inflammation. A multiplicative approach was selected to capture the biological premise that these two pathways may interact synergistically, such that patients with simultaneous elevations in both metabolic and inflammatory burden may experience greater risk than would be expected from either pathway alone. Similar combined TyG–SII approaches have been evaluated in prior cardiovascular and population-based studies, supporting the integration of metabolic and inflammatory information into a single composite biomarker (8, 9). However, this specific composite has not been established as a validated clinical risk score in the TAVR population. Therefore, TSI was evaluated as an exploratory, hypothesis-generating biomarker rather than as an established prognostic score. For analysis, TSI was treated as a continuous variable. To facilitate interpretation of effect sizes, TSI values were standardized to z-scores, with hazard ratios reported per one standard deviation increase in TSI.

### 2.3 Echocardiographic follow-up

Patients underwent systematic follow-up with transthoracic echocardiography (TTE) at approximately 6 months, 1 year, and annually thereafter following TAVR, in addition to baseline and immediate post-procedural assessments. All examinations were performed by experienced cardiologists and included standardized evaluation of bioprosthetic valve function, encompassing mean transvalvular gradient, peak velocity, effective orifice area, Doppler velocity index, and assessment of both intraprosthetic and paravalvular aortic regurgitation. These follow-up intervals were selected to capture both early and long-term changes in valve performance. When scheduled follow-up examinations were unavailable, echocardiographic data were obtained at the next clinical visit or retrieved from outside institutions. Clinically indicated adjunctive imaging, including transesophageal echocardiography and multi-slice computed tomography, was also reviewed when available to assess leaflet abnormalities or suspected valve thrombosis.

### 2.4 Outcomes

To reduce confounding between LVEF groups, we constructed an exact matched cohort using 1:4 stratified matching. Patients with LVEF ≤ 40% (“cases”) were each matched to up to four patients with LVEF > 40% (“controls”) of the same sex, age category, TAVR calendar period, and prosthesis type (self-expandable vs. balloon-expandable valve). This yielded a matched subset of 646 patients (LVEF ≤ 40% cases and LVEF > 40% matched controls) for primary analyses. The primary outcome was long-term all-cause mortality, ascertained from hospital records and national death indices. The secondary outcome was bioprosthetic valve failure manifesting as hemodynamic valve deterioration (HVD), defined according to Valve Academic Research Consortium-3 criteria (VARC-3) as a clinically relevant worsening of transprosthetic valve performance on follow-up echocardiography, including any of the following: an increase in mean transvalvular gradient of >10 mmHg reaching an absolute value ≥ 20 mmHg, a reduction in effective orifice area (EOA) of ≥0.3 cm^2^, a decrease in Doppler velocity index (DVI) of ≥0.1, or the development of new or worsening transvalvular aortic regurgitation by at least one grade resulting in moderate or greater regurgitation (16, 17).

### 2.5 Statistical analysis

All analyses were conducted using Stata 17.0 (StataCorp, College Station, TX). Continuous variables are presented as mean ± standard deviation or median (Q1–Q3) as appropriate, and categorical variables as frequencies and percentages. Group for baseline characteristics were performed using Student's *t*-test or Wilcoxon rank-sum test for continuous data, and chi-square or Fisher's exact tests for categorical data.

Time-to-event analyses were performed using Cox proportional hazards regression to estimate hazard ratios (HRs) and 95% confidence intervals (CIs) comparing reduced LVEF (≤40%) vs. LVEF > 40%. To account for within-stratum correlation induced by the matching process, robust standard errors were computed by clustering on the matching stratum identifier.

Time-to-event analysis for long-term all-cause mortality was performed using Cox proportional hazards regression. Analyses were conducted separately within each LVEF stratum (≤40% and >40%). The primary exposure, standardized TSI (z-score, per 1-SD increase), was forced into all models. Covariate selection followed a combined clinical and statistical approach. First, a set of clinically relevant candidate variables, including demographic characteristics, major comorbidities, and key laboratory and procedural variables reflecting baseline risk and disease severity, was prespecified based on prior literature and clinical relevance (2, 7). Second, these candidate variables were evaluated in univariable Cox regression analyses, and variables demonstrating evidence of association with mortality (typically *P* < 0.10) and/or established clinical importance were considered for multivariable adjustment. Given the relatively limited number of mortality events in the LVEF ≤ 40% subgroup, multivariable modeling was intentionally kept parsimonious to reduce the risk of overfitting. Accordingly, the number of covariates included in the final models was constrained according to the events-per-variable (EPV) principle, maintaining approximately 10 outcome events per estimated parameter in the LVEF ≤ 40% subgroup. This resulted in a simplified adjusted model for the LVEF ≤ 40% subgroup and a more comprehensive model for the LVEF > 40% subgroup, where the larger number of outcome events permitted additional adjustment. Hazard ratios (HRs) with 95% confidence intervals (CIs) are reported.

Long-term HVD was analyzed using Cox proportional hazards regression in a 1:4 time-dependent matched cohort, restricted to matched observations. The primary exposure was left ventricular ejection fraction (LVEF ≤ 40% vs. >40%). Multivariable adjustment was performed using a clinically driven and statistically informed approach. A limited set of covariates was selected *a priori* based on known associations with valve durability and structural valve deterioration (18, 19) including demographic factors, metabolic comorbidity, renal function, and procedural characteristics. To avoid overfitting, the number of covariates was constrained according to an events-per-variable principle (∼10 events per parameter). Hazard ratios (HRs) with 95% confidence intervals (CIs) are reported. The proportional hazards assumption was assessed using Schoenfeld residuals and was not violated for the final models.

To formally assess whether baseline LVEF modified the association between standardized TSI and long-term all-cause mortality, we fitted an additional multivariable Cox proportional hazards model in the overall mortality cohort (*n* = 646). The model included standardized TSI, LVEF group (≤40% vs. >40%), and their multiplicative interaction term, with adjustment for age, sex, atrial fibrillation, and serum creatinine. Statistical significance of the interaction was assessed using the Wald test, and stratum-specific hazard ratios for each 1-SD increase in standardized TSI were estimated from the fitted interaction model.

For the secondary analysis of long-term hemodynamic valve deterioration, a separate 1:4 time-dependent matched cohort comprising 224 patients with 63 HVD events was analyzed. The primary adjusted Cox model included LVEF group, age, sex, serum creatinine, and valve size. In an additional exploratory analysis within the same time-dependent matched cohort, we assessed whether the association between standardized TSI and HVD differed according to baseline LVEF by including standardized TSI, LVEF group, and their multiplicative interaction term, with adjustment for age, sex, serum creatinine, and valve size. The Wald test was used to evaluate the standardized TSI × LVEF interaction, and stratum-specific hazard ratios were estimated from the interaction model.

To improve transparency, the mortality and HVD analyses were conducted independently within their respective matched cohorts. Detailed multivariable Cox regression models for each analysis are provided in Supplementary eTables 1–3.

Among patients with LVEF ≤ 40%, the addition of standardized TSI to the base clinical model modestly improved model discrimination, with Harrell's C-index increasing from 0.616 to 0.629. The extended model demonstrated significantly better overall fit than the base model according to the likelihood-ratio test (LR *χ*^2^ = 6.49; *P* = 0.011). The addition of standardized TSI was also associated with lower Akaike information criterion (AIC) and Bayesian information criterion (BIC) values compared with the base model (AIC, 339.47 vs. 343.96; BIC, 354.62 vs. 356.08), supporting an incremental prognostic contribution of TSI beyond the selected clinical covariates (Supplementary eTable 4).

Missing data were minimal (<5% across all variables). Continuous covariates with missing values were imputed using mean imputation, whereas categorical covariates with missing values were imputed using mode imputation. Complete-case analysis was used for the primary exposure and primary outcome variables; therefore, no exposure or outcome data were imputed. All tests were two-sided, and a *p* value < 0.05 was considered statistically significant.

## 3 Results

Baseline characteristics according to LVEF are summarized in Table 1. Patients with LVEF ≤ 40% were slightly younger than those with LVEF > 40% (79.5 ± 8.8 vs. 81.0 ± 7.1 years, *P* = 0.036) and had a lower body mass index (27.1 ± 4.7 vs. 28.5 ± 5.0 kg/m^2^, *P* = 0.002). The reduced LVEF group showed a higher burden of cardiovascular comorbidities, including atrial fibrillation (35.3% vs. 26.6%, *P* = 0.037), coronary artery disease (58.8% vs. 48.9%, *P* = 0.032), and peripheral vascular disease (19.0% vs. 11.8%, *P* = 0.023).

**Table 1:** Baseline characteristics according to left ventricular ejection fraction (LVEF ≤ 40% vs. >40%) in a 1:4 case-control matched cohort (*n* = 646), matched on sex, age category, calendar period of TAVR, and valve type.

Variable	Category	LVEF ≤ 40% arm	LVEF > 40% arm	*P* value
		*n* = 153 (%)	*n* = 493 (%)	
Demographic data				
Age, y	Mean ± SD	79.501 ± 8.778	80.964 ± 7.079	0.036^a^
Gender	Female, *n* (%)	56 (36.6)	208 (42.2)	0.219^b^
BMI, kg/m^2^	Mean ± SD	27.057 ± 4.650	28.507 ± 5.016	0.002^a^
STS Score	Mean ± SD	7.96 ± 2.23	6.96 ± 1.53	0.236^a^
Medical History				
Diabetes Mellitus	Yes, *n* (%)	69 (45.1)	220 (44.6)	0.918^b^
Hypertension	Yes, *n* (%)	128 (83.7)	427 (86.6)	0.359^b^
Smoker	Yes, *n* (%)	30 (19.6)	77 (15.6)	0.246^b^
Dyslipidemia	Yes, *n* (%)	121 (79.1)	393 (79.7)	0.866^b^
Atrial fibrillation	Yes, *n* (%)	54 (35.3)	131 (26.6)	0.037^b^
CAD	Yes, *n* (%)	90 (58.8)	241 (48.9)	0.032^b^
PVD	Yes, *n* (%)	29 (19.0)	58 (11.8)	0.023^b^
Laboratory				
White blood cells (K/uL)	Mean ± SD	7.192 ± 2.531	7.508 ± 3.470	0.296^a^
Platelets (K/uL)	Mean ± SD	253.425 ± 52.436	244.442 ± 49.888	0.055^a^
Total cholesterol (mg/dl)	Mean ± SD	145.320 ± 37.601	154.964 ± 37.783	0.006^a^
Albumin (g/dl)	Mean ± SD	3.843 ± 0.389	3.963 ± 0.363	0.001^a^
Creatinine (mg/dl)	Mean ± SD	1.442 ± 1.167	1.224 ± 0.898	0.015^a^
Standardized values of TSI (z-score)	Mean ± SD	0.411 ± 1.229	−0.127 ± 0.881	<0.001^a^
Echocardiography Pre-Procedural				
Septum thickness (mm)	Mean ± SD	12.307 ± 1.994	12.833 ± 2.009	0.005^a^
LVeDD (mm)	Mean ± SD	51.190 ± 5.932	44.843 ± 5.292	<0.001^a^
Aortic valve Mean gradient (mm Hg)	Mean ± SD	43.577 ± 12.267	45.326 ± 12.527	0.130^a^
Aortic valve area (cm^2^)	Mean ± SD	0.638 ± 0.164	0.724 ± 0.176	<0.001^a^
Echocardiography post-procedural				
Septum thickness (mm)	Mean ± SD	12.842 ± 3.411	13.107 ± 2.137	0.252^a^
LVeDD (mm)	Mean ± SD	49.993 ± 6.597	44.285 ± 5.224	<0.001^a^
Aortic valve Mean gradient (mm Hg)	Mean ± SD	9.241 ± 2.874	9.351 ± 3.095	0.696^a^
Procedural parameters				
Valve type	Self-expandable	89 (58.2)	318 (64.5)	0.156^b^
Valve size (mm)	Mean ± SD	27.871 ± 2.711	26.974 ± 2.682	<0.001^a^
Post dilatation	Yes	39 (25.5)	140 (28.4)	0.483^b^
Procedural duration (minutes)	Mean ± SD	78.071 ± 30.533	79.592 ± 29.941	0.585^a^
Contrast medium volume (mL)	Mean ± SD	110.010 ± 49.857	112.466 ± 55.975	0.627^a^
Electrocardiography				
PR Interval (ms)	Mean ± SD	188.732 ± 23.794	187.265 ± 24.408	0.514^a^
QTc Interval (ms)	Mean ± SD	447.937 ± 40.740	440.107 ± 21.587	0.002^a^
QRS Duration (ms)	Mean ± SD	114.912 ± 25.740	106.585 ± 18.958	<0.001^a^
CTA characteristics Pre-Procedural				
Aortic valve calcium score	Mean ± SD	2,426.275 ± 974.955	2,491.808 ± 1,078.478	0.502^a^
LVOT calcification	Yes, *n* (%)	37 (24.2)	133 (27.0)	0.904^b^
Annular geometry Pre- Procedural				
Annulus area (mm^2^)	Mean ± SD	480.255 ± 87.128	451.877 ± 76.847	<0.001^a^
Effective annulus diameter (area-derived, mm)	Mean ± SD	24.692 ± 1.974	23.935 ± 1.854	<0.001^a^
Valve orientation				
Annulus angulation (degrees)	Mean ± SD	47.024 ± 6.605	47.137 ± 6.088	0.843^a^
Access vessels				
Minimal access vessel diameter (mm)	Mean ± SD	6.843 ± 1.167	7.063 ± 1.147	0.039^a^

Values are presented as mean ± standard deviation or *n* (%), as appropriate.

a *P* values were calculated using the Student's t-test.

b *P* values were calculated using the *χ*^2^ test.

LVEF, left ventricular ejection fraction; TAVR, transcatheter aortic valve replacement; TSI, a composite inflammatory–metabolic index derived from the triglyceride–glucose (TyG) index and the systemic immune-inflammation index (SII).; BMI, body mass index; STS, Society of Thoracic Surgeons; CAD, coronary artery disease; PVD, peripheral vascular disease; AF, atrial fibrillation; CTA, computed tomography angiography; WBC, white blood cell count; LVeDD, left ventricular end-diastolic diameter; LVOT, left ventricular outflow tract; HVD, hemodynamic valve deterioration; HR, hazard ratio; CI, confidence interval; SD, standard deviation.

Laboratory findings demonstrated higher creatinine levels (1.44 ± 1.17 vs. 1.22 ± 0.90 mg/dL, *P* = 0.015) and lower albumin concentrations (3.84 ± 0.39 vs. 3.96 ± 0.36 g/dL, *P* = 0.001) in patients with LVEF ≤ 40%. Importantly, standardized TSI values were significantly higher in the reduced LVEF group (0.41 ± 1.23 vs. −0.13 ± 0.88, *P* < 0.001). Echocardiographic assessment showed larger left ventricular dimensions and smaller aortic valve area in patients with reduced LVEF, both before and after the procedure (all *P* < 0.001).

During follow-up, 51 deaths (33.3%) occurred in the LVEF ≤ 40% group and 136 deaths (27.6%) in the LVEF > 40% group. Univariable Cox analyses are shown in Table 2. In both LVEF strata, atrial fibrillation and renal dysfunction were consistently associated with higher mortality (LVEF ≤ 40%: AF HR 1.91, *P* = 0.029; creatinine HR 1.29, *P* = 0.029; LVEF > 40%: AF HR 1.81, *P* = 0.001; creatinine HR 1.27, *P* = 0.004). In the LVEF > 40% group, hypoalbuminemia was a strong predictor of mortality (HR 0.33, *P* < 0.001), whereas post-dilatation was associated with increased risk (HR 1.89, *P* = 0.001).

**Table 2:** Long-term all-cause mortality in a 1:4 case-control matched cohort (*n* = 646), matched on sex, age category, calendar period of TAVR, and valve type, stratified by LVEF (≤40% vs. >40%).

		Long-term all-cause mortality			
Variable	Reference	LVEF ≤ 40% arm		LVEF > 40% arm	
		*n* = 153		*n* = 493	
		51 (33.33%) events		136 (27.59%) events	
		HR (95% CI)	*P* value	HR (95% CI)	*P* value
Demographic data					
Age, y	per 1-unit increase	1.021 (0.975–1.069)	0.382	1.009 (0.980–1.038)	0.546
Gender	Male, *n* (%)	0.768 (0.432–1.366)	0.369	1.612 (1.135–2.288)	0.008
BMI, kg/m^2^	per 1-unit increase	1.009 (0.950–1.072)	0.767	0.974 (0.942–1.007)	0.123
Medical History					
Diabetes Mellitus	No, *n* (%)	1.668 (0.940–2.958)	0.080	1.064 (0.756–1.497)	0.724
Hypertension	No, *n* (%)	1.950 (0.597–6.373)	0.269	1.358 (0.623–2.959)	0.441
Smoker	No, *n* (%)	0.905 (0.449–1.827)	0.782	1.322 (0.828–2.110)	0.243
Dyslipidemia	No, *n* (%)	1.747 (0.885–3.449)	0.108	1.109 (0.745–1.652)	0.611
Atrial fibrillation	No, *n* (%)	1.907 (1.069–3.404)	0.029	1.805 (1.275–2.556)	0.001
CAD	No, *n* (%)	0.891 (0.509–1.559)	0.685	1.079 (0.763–1.527)	0.665
PVD	No, *n* (%)	0.930 (0.433–1.998)	0.852	1.404 (0.878–2.244)	0.156
Laboratory					
White blood cells (K/uL)	per 1-unit increase	1.045 (0.946–1.156)	0.385	1.030 (0.982–1.079)	0.226
Platelets (K/uL)	per 1-unit increase	0.997 (0.992–1.002)	0.248	1.001 (0.997–1.004)	0.736
Total cholesterol (mg/dl)	per 1-unit increase	0.998 (0.991–1.005)	0.604	0.997 (0.992–1.001)	0.137
Albumin (g/dl)	per 1-unit increase	0.700 (0.362–1.357)	0.291	0.334 (0.194–0.576)	<0.001
Creatinine (mg/dl)	per 1-unit increase	1.294 (1.027–1.629)	0.029	1.268 (1.077–1.493)	0.004
Echocardiography Pre-Procedural					
Septum thickness (mm)	per 1-unit increase	1.003 (0.862–1.168)	0.965	1.008 (0.925–1.099)	0.853
LVeDD (mm)	per 1-unit increase	1.014 (0.966–1.065)	0.565	0.994 (0.961–1.028)	0.731
Aortic valve Mean gradient					
(mm Hg)	per 1-unit increase	1.011 (0.986–1.036)	0.410	0.993 (0.980–1.006)	0.309
Aortic valve area (cm^2^)	per 1-unit increase	1.137 (0.208–6.207)	0.882	0.613 (0.217–1.729)	0.355
Echocardiography post-procedural					
Septum thickness (mm)	per 1-unit increase	1.047 (0.990–1.108)	0.106	1.009 (0.923–1.104)	0.842
LVeDD (mm)	per 1-unit increase	1.003 (0.959–1.050)	0.887	1.000 (0.969–1.032)	0.999
Aortic valve Mean gradient					
(mm Hg)	per 1-unit increase	0.931 (0.860–1.007)	0.076	1.050 (1.000–1.102)	0.048
Procedural parameters					
Valve type	Balloon-expandable vs. Self-expandable	1.089 (0.585–2.025)	0.788	1.016 (0.702–1.471)	0.932
Valve size (mm)	–	0.995 (0.902–1.098)	0.920	1.073 (0.999–1.152)	0.052
Post dilatation	No, *n* (%)	1.579 (0.824–3.027)	0.169	1.885 (1.282–2.771)	0.001
Procedural duration (minutes)	per 1-unit increase	1.009 (1.000–1.019)	0.057	1.005 (0.999–1.011)	0.084
Contrast medium volume (mL)	per 1-unit increase	0.998 (0.992–1.004)	0.503	0.999 (0.997–1.002)	0.635
Electrocardiography					
PR Interval (ms)	per 1-unit increase	1.000 (0.991–1.009)	0.965	1.000 (0.995–1.006)	0.958
QTc Interval (ms)	per 1-unit increase	0.998 (0.991–1.004)	0.496	1.004 (0.998–1.011)	0.226
QRS Duration (ms)	per 1-unit increase	0.997 (0.989–1.005)	0.520	1.006 (0.999–1.013)	0.117
CTA characteristics Pre-Procedural					
Aortic valve calcium score	per 1-unit increase	1.000 (1.000–1.001)	0.231	1.000 (1.000–1.000)	0.715
LVOT calcification	No, *n* (%)	0.969 (0.364–2.576)	0.949	1.350 (0.718–2.540)	0.352
Annular geometry Pre- Procedural					
Annulus area (mm^2^)	per 1-unit increase	1.002 (0.998–1.006)	0.369	1.003 (1.000–1.006)	0.023
Effective annulus diameter (area-derived, mm)	per 1-unit increase	1.068 (0.888–1.284)	0.485	1.126 (0.998–1.269)	0.054
Valve orientation					
Annulus angulation (degrees)	per 1-unit increase	1.063 (0.992–1.139)	0.086	1.038 (0.990–1.088)	0.125
Access vessels					
Minimal access vessel diameter (mm)	per 1-unit increase	0.947 (0.672–1.335)	0.757	0.960 (0.804–1.145)	0.647

HR, hazard ratio; CI, confidence interval; LVEF, left ventricular ejection fraction; TAVR, transcatheter aortic valve replacement.

**Figure 2:**
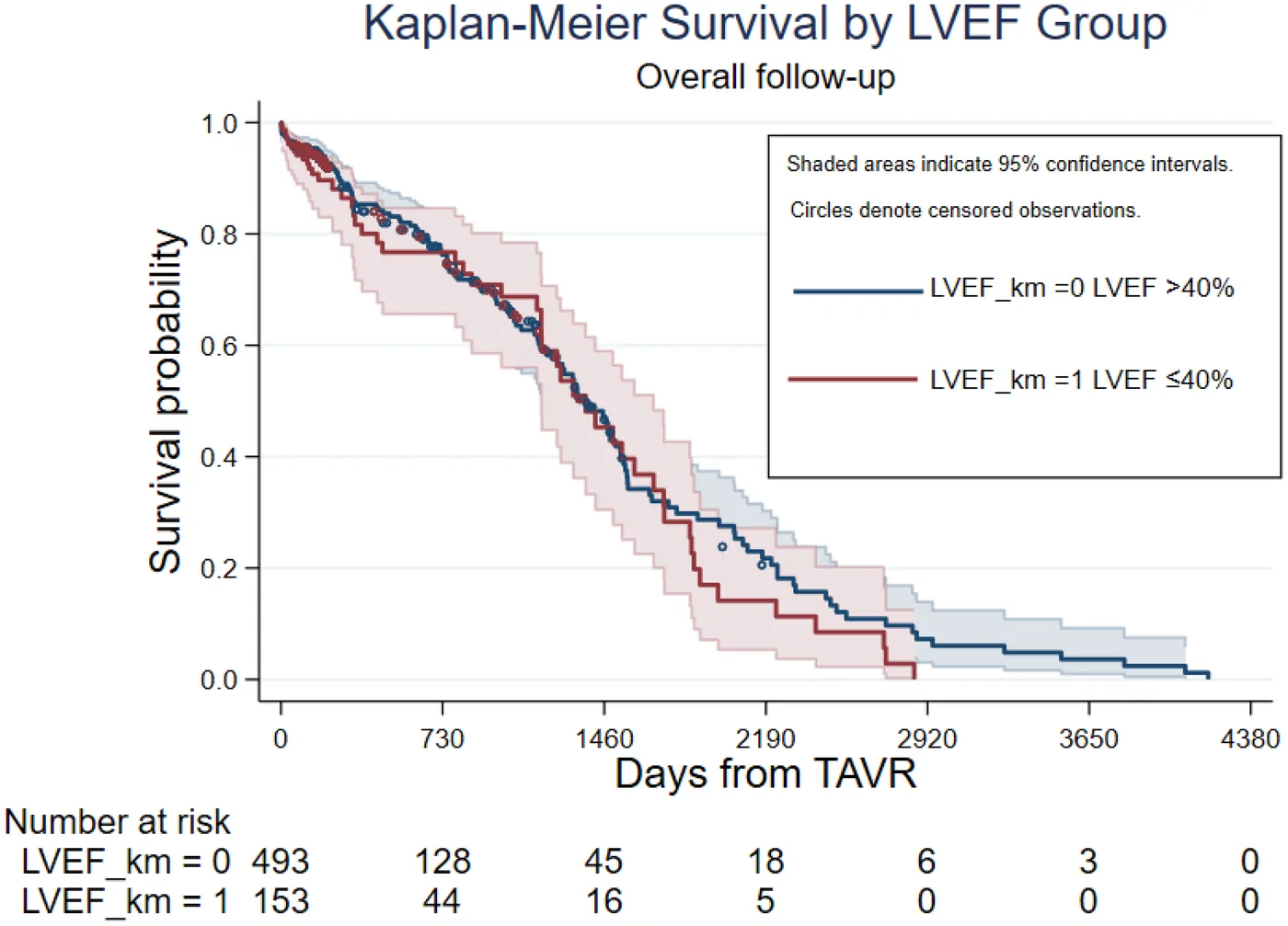
Kaplan–Meier survival curves for long-term all-cause mortality stratified by LVEF group (≤40% vs. >40%) in the 1:4 matched cohort (*n* = 646). Shaded areas represent 95% confidence intervals. Circles indicate censored observations. Numbers at risk are shown below the plot. Group comparison was performed using the log-rank test.

Figure 2 shows Kaplan–Meier survival curves stratified by LVEF group in the 1:4 matched cohort (*n* = 646). All-cause mortality occurred in 51/153 patients (33.3%) with LVEF ≤ 40% and in 136/493 patients (27.6%) with LVEF > 40%. There was no statistically significant difference in survival between groups [log-rank *χ*^2^ (1) = 1.47, *p* = 0.2248]. In unadjusted Cox regression, LVEF ≤ 40% was not significantly associated with all-cause mortality (HR, 1.22, 95% CI, 0.88–1.69; *p* = 0.226).

As shown in Table 3, higher standardized TSI was associated with increased mortality in patients with **LVEF** ≤ **40%** in the crude analysis (HR, 1.38, 95% CI, 1.12–1.70, *P* = 0.003). This association remained **independent after multivariable adjustment** for key clinical confounders (adjusted HR, 1.38, 95% CI, 1.10–1.73, *P* = 0.005). In contrast, no significant association between TSI and mortality was observed in patients with **LVEF** > **40%**, either before or after adjustment (adjusted HR, 1.02, 95% CI, 0.84–1.24, *P* = 0.818). **Detailed multivariable Cox regression analyses evaluating the association between standardized TSI and long-term all-cause mortality stratified by LVEF are provided in** Supplementary eTables 1–2.

**Table 3:** Association between standardized TSI (z-score) and long-term all-cause mortality in the overall cohort (*n* = 646), stratified by baseline LVEF (≤40% vs. >40%).

		Long-term all-cause mortality			
		LVEF ≤ 40% arm^a^		LVEF > 40% arm^b^	
		*n* = 153		*n* = 493	
		51 (33.33%) events		136 (27.59%) events	
Variable	Reference	HR (95% CI)	*P* value	HR (95% CI)	*P* value
Crude					
Standardized values of TSI (z-score)	per 1-SD increase	1.379 (1.118–1.701)	0.003	1.135 (0.936–1.376)	0.198
Adjusted					
Standardized values of TSI (z-score)	per 1-SD increase	1.379 (1.102–1.726)	0.005	1.023 (0.844–1.239)	0.818

a Adjusted for age, sex, atrial fibrillation, and creatinine, in addition to standardized TSI (z-score).

b Adjusted for age, sex, body mass index, atrial fibrillation, albumin, creatinine, post-dilatation, and annulus area, in addition to standardized TSI (z-score). The proportional hazards assumption was not violated.

TSI, a composite inflammatory–metabolic index derived from the triglyceride–glucose (TyG) index and the systemic immune-inflammation index (SII).; HR, hazard ratio; CI, confidence interval; LVEF, left ventricular ejection fraction.

**Figure 3:**
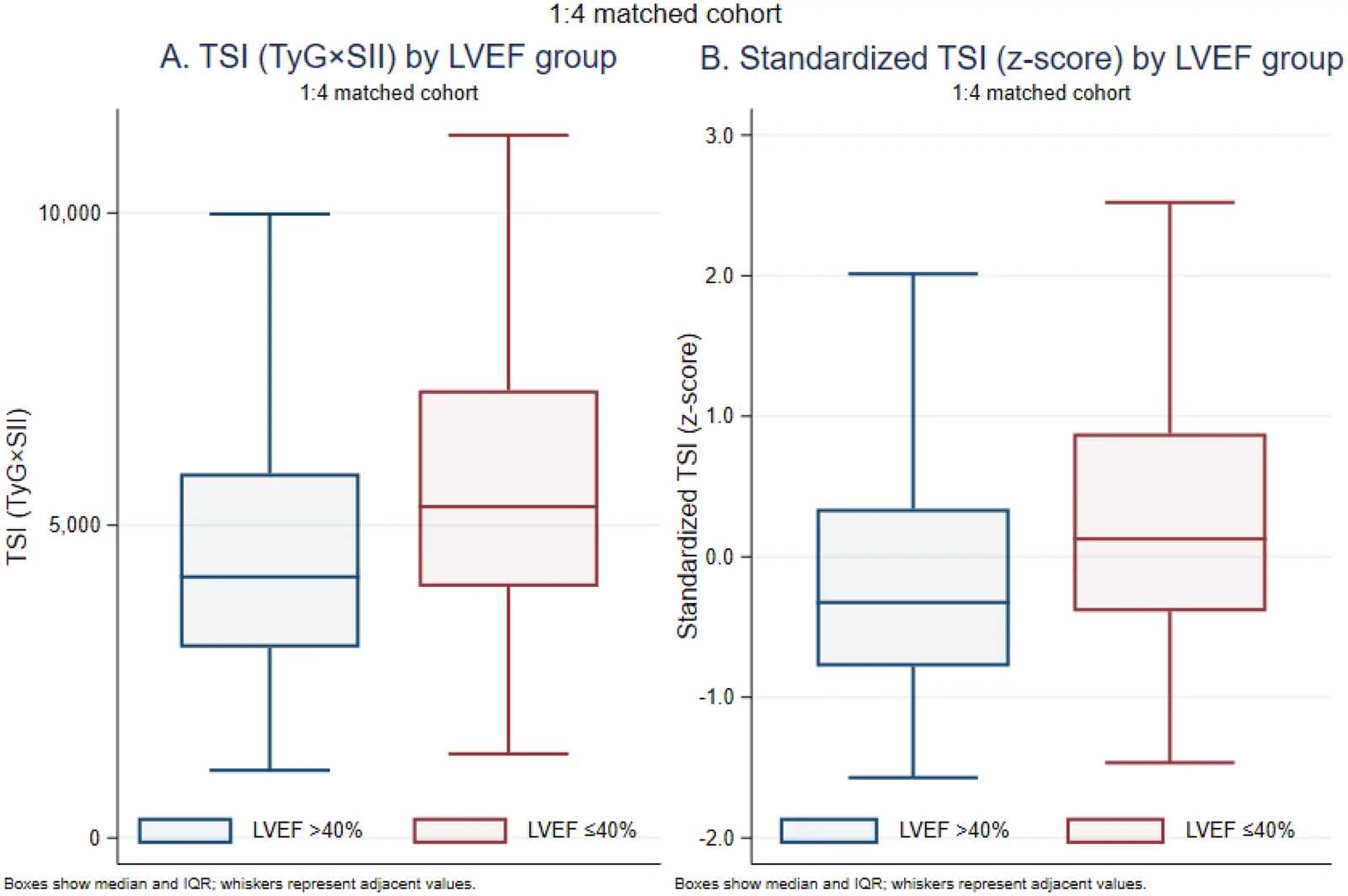
Distribution of TSI measures by LVEF group. Distribution of triglyceride -glucose index combined with systemic immune-inflammation index [TSI (TyG × SII)] and standardized TSI (z-score) according to left ventricular ejection fraction (LVEF) groups in a 1:4 matched cohort. (**A**) Distribution of TSI (TyG × SII). (**B**) Distribution of standardized TSI (z-score). Boxes represent the median and interquartile range (IQR), and whiskers denote adjacent values. Patients with reduced LVEF (≤40%) demonstrated a right-shifted distribution for both TSI measures compared with those with LVEF >40%.

In the 1:4 matched cohort, patients with reduced left ventricular ejection fraction (LVEF ≤ 40%) had higher TSI values than those with LVEF > 40%. As shown in Figure 3A, the distribution of TSI (TyG × SII) was shifted toward higher values in the LVEF ≤ 40% group, with a higher median and wider interquartile range. Consistent findings were observed for standardized TSI (z-score) (Figure 3B), indicating a higher inflammatory-metabolic burden among patients with impaired systolic function.

In the time-dependent matched cohort (Table 4), long-term HVD occurred in 63 patients (28.1%). Reduced LVEF was associated with a higher risk of HVD in both the crude model (HR, 1.76, 95% CI, 1.05–2.95, *P* = 0.034) and the adjusted model (HR, 1.71, 95% CI, 1.00–2.91, *P* = 0.048). However, given the relatively small matched cohort and the borderline statistical significance of the adjusted association, these findings should be interpreted cautiously and considered hypothesis-generating. Detailed multivariable Cox regression analyses evaluating the association between standardized TSI and long-term all-cause mortality within each LVEF stratum are presented in Supplementary eTables 1–2, while the fully adjusted model for long-term HVD is provided in Supplementary eTable 3.

**Table 4:** Association between left ventricular ejection fraction (LVEF ≤ 40% vs. >40%) and long-term hemodynamic valve deterioration (HVD) in a 1:4 time-dependent matched cohort (*n* = 224), matched on sex, age category, calendar period of TAVR, and valve type.

		Long-term HVD^a^	
		*n* = 224	
		63 (28.13%) events	
Variable	Reference	HR (95% CI)	*P* value
Crude			
LVEF > 40% arm	Ref	–	–
LVEF ≤ 40% arm	–	1.755 (1.045–2.947)	0.034
Adjusted			
LVEF > 40% arm	Ref	–	–
LVEF ≤ 40% arm	–	1.710 (1.004–2.912)	0.048

a Adjusted Cox model included age, sex, creatinine, and valve size in addition to LVEF. The proportional hazards assumption was not violated.

LVEF, left ventricular ejection fraction; HVD, hemodynamic valve deterioration; HR, hazard ratio; CI, confidence interval.

**Figure 4:**
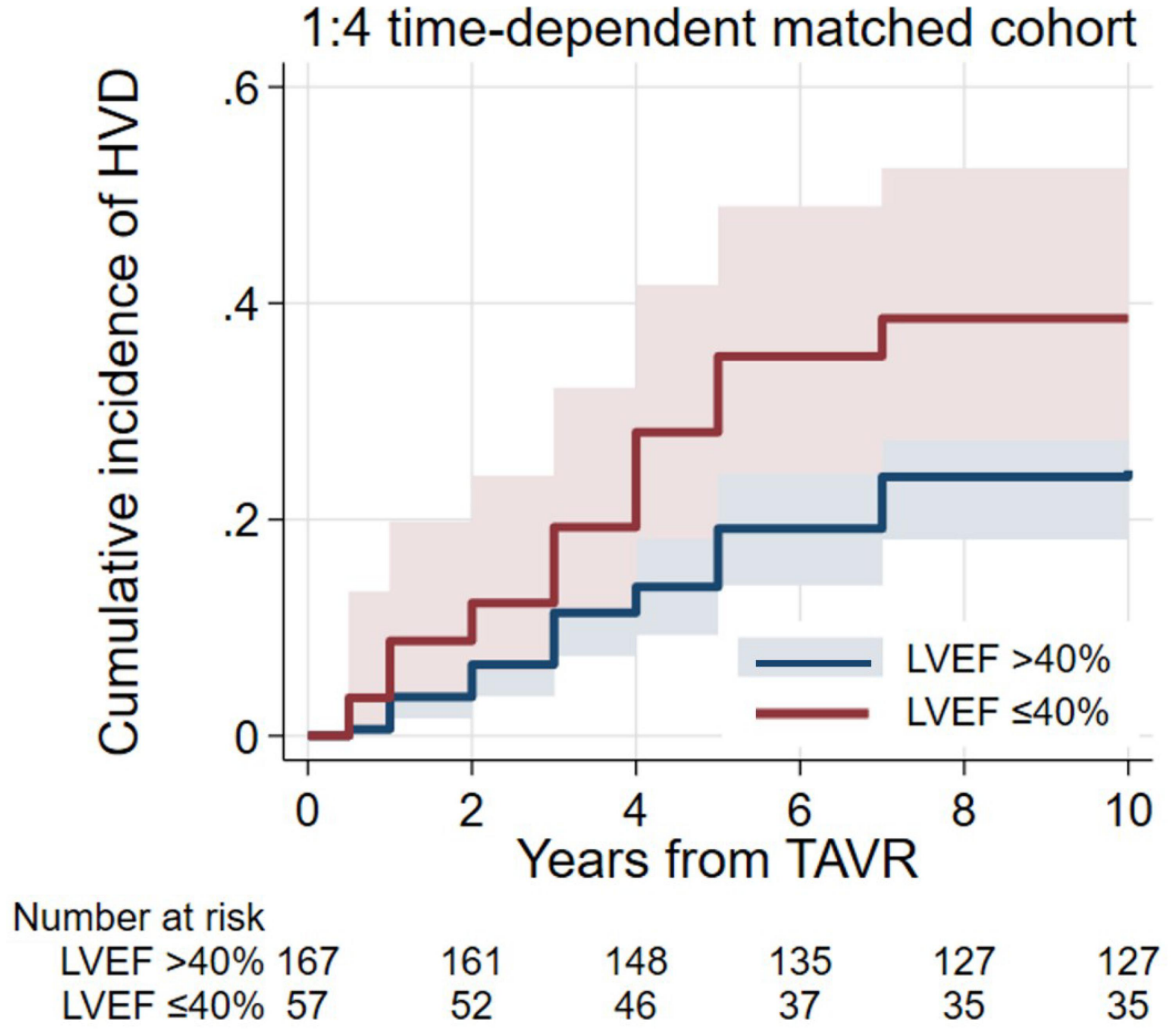
Cumulative incidence of hemodynamic valve deterioration by left ventricular ejection fraction. Cumulative incidence curves for hemodynamic valve deterioration (HVD) stratified by baseline left ventricular ejection fraction (LVEF) in a 1:4 time-dependent matched cohort. Patients with reduced LVEF (≤40%) demonstrated a consistently higher cumulative incidence of HVD over long-term follow-up compared with those with LVEF > 40%. Shaded areas represent 95% confidence intervals. Numbers at risk are displayed below the plot.

In the time-dependent matched cohort, patients with reduced LVEF (≤40%) exhibited a higher cumulative incidence of hemodynamic valve deterioration throughout follow-up compared with those with LVEF > 40% (Figure 4). The divergence between groups emerged early after TAVR and progressively widened over time, indicating an increased susceptibility to valve hemodynamic deterioration in the setting of impaired left ventricular systolic function.

Formal interaction analyses were performed to evaluate whether baseline LVEF modified the association between standardized TSI and long-term outcomes (Table 5). For long-term all-cause mortality, the standardized TSI × LVEF interaction was statistically significant (*P* for interaction = 0.007), indicating that the prognostic association of TSI differed according to baseline left ventricular systolic function. Each 1-SD increase in standardized TSI was associated with a higher risk of mortality among patients with LVEF ≤ 40% (HR, 1.39, 95% CI, 1.12–1.73; *P* = 0.003), whereas no significant association was observed among patients with LVEF > 40% (HR, 1.08, 95% CI, 0.89–1.30; *P* = 0.455). In the time-dependent matched HVD cohort, each 1-SD increase in standardized TSI was similarly associated with a higher risk of HVD among patients with LVEF ≤ 40% (HR, 1.53, 95% CI, 1.07–2.19; *P* = 0.020), but not among patients with LVEF > 40% (HR, 1.10, 95% CI, 0.75–1.62; *P* = 0.623).

**Table 5:** Interaction between standardized TSI and baseline left ventricular ejection fraction for long-term all-cause mortality and hemodynamic valve deterioration.

Outcome	Analysis cohort	LVEF group	HR per 1-SD increase in standardized TSI (95% CI)	*P* value	*P* for interaction
Long-term all-cause mortality	Overall cohort, *n* = 646; 187 events 1:4 time-dependent matched cohort, *n* = 224; 63 events	LVEF > 40%	1.075 (0.889–1.300)	0.455	0.007
		LVEF ≤ 40%	1.393 (1.122–1.728)	0.003	
Long-term HVD		LVEF > 40%	1.101 (0.749–1.619)	0.623	0.211
		LVEF ≤ 40%	1.533 (1.071–2.195)	0.020	

a The mortality interaction model included standardized TSI, LVEF group, the standardized TSI × LVEF interaction term, age, sex, atrial fibrillation, and serum creatinine.

b The HVD interaction model was performed in the 1:4 time-dependent matched cohort and included standardized TSI, LVEF group, the standardized TSI × LVEF interaction term, age, sex, serum creatinine, and valve size.

c Hazard ratios represent the association with each 1-standard deviation increase in standardized TSI within each LVEF group.

d *P* values for interaction were obtained from Wald tests of the standardized TSI × LVEF interaction terms.

CI, confidence interval; HR, hazard ratio; HVD, hemodynamic valve deterioration; LVEF, left ventricular ejection fraction; TSI, TyG–SII composite index; TyG, triglyceride–glucose index; SII, systemic immune-inflammation index.

Among patients with LVEF ≤ 40%, the addition of standardized TSI to the base clinical model modestly improved model discrimination, with Harrell's C-index increasing from 0.616 to 0.629. The extended model demonstrated significantly better overall fit than the base model according to the likelihood-ratio test (LR *χ*^2^ = 6.49; *P* = 0.011). The addition of standardized TSI was also associated with lower AIC and BIC values compared with the base model (AIC, 339.47 vs. 343.96; BIC, 354.62 vs. 356.08), supporting an incremental prognostic contribution of TSI beyond the selected clinical covariates (Supplementary eTable 4).

## 4 Discussion

In this matched cohort of patients undergoing TAVR, we demonstrate that a heightened inflammatory–metabolic burden, quantified by the TSI, is associated with increased long-term mortality exclusively among patients with reduced left ventricular systolic function (LVEF ≤ 40%). Importantly, baseline LVEF alone was not independently associated with mortality after rigorous matching for demographic and procedural factors, underscoring that ventricular dysfunction *per se* does not fully capture post-TAVR risk. Rather, our findings suggest that myocardial vulnerability modulates the prognostic impact of systemic inflammation and metabolic dysregulation, rendering patients with impaired systolic function particularly susceptible to their adverse effects. In parallel, reduced LVEF was associated with a higher risk of long-term hemodynamic valve deterioration in the smaller time-dependent matched cohort, even after adjustment for host- and valve-related factors. However, given the limited sample size and the borderline statistical significance of the adjusted association, this finding should be interpreted cautiously and requires confirmation in larger prospective studies. Collectively, these results indicate that adverse outcomes after TAVR are driven not solely by baseline ventricular function, but by its interaction with systemic inflammatory–metabolic stress and long-term valve performance.

The formal interaction analysis demonstrated that baseline LVEF significantly modified the association between standardized TSI and long-term all-cause mortality. Higher standardized TSI was associated with an increased risk of mortality among patients with LVEF ≤ 40%, whereas no significant association was observed among those with LVEF > 40%. This finding supports the concept that the prognostic relevance of inflammatory–metabolic burden may depend on the degree of underlying left ventricular systolic dysfunction. Patients with reduced LVEF may have diminished myocardial reserve, greater neurohormonal activation, increased wall stress, and a reduced capacity to tolerate systemic inflammatory and metabolic disturbances. Accordingly, a higher TSI may identify a particularly vulnerable subgroup in whom adverse systemic biological pathways translate more strongly into long-term mortality after TAVR. In contrast, although standardized TSI was associated with HVD among patients with reduced LVEF, the formal interaction was not statistically significant for this outcome. Therefore, the HVD findings should be interpreted as exploratory and should not be considered definitive evidence of effect modification.

Beyond its independent association with mortality in patients with reduced LVEF, standardized TSI provided incremental prognostic information when added to a parsimonious clinical model. The addition of TSI modestly increased Harrell's C-index and significantly improved overall model fit according to the likelihood-ratio test, with corresponding reductions in both AIC and BIC. These findings suggest that TSI captures prognostic information not fully represented by age, sex, atrial fibrillation, and renal function. Nevertheless, the absolute improvement in discrimination was modest, and the difference between C-index values was not formally tested. Therefore, these results support the potential complementary prognostic value of TSI rather than establishing substantial or clinically definitive improvement in risk prediction.

One possible explanation for the observed variation in the association according to systolic function is that a metabolically inflamed milieu becomes clinically consequential when myocardial reserve is limited. In patients with LVEF ≤ 40%, reduced contractile reserve, increased wall stress, and neurohormonal activation may amplify the adverse effects of insulin resistance–related metabolic dysfunction and systemic inflammation, thereby increasing vulnerability to periprocedural stressors, including rapid pacing, transient hypotension, and contrast exposure, as well as to persistent maladaptive remodeling after ventricular unloading. Contemporary mechanistic studies further suggest that TAVR induces a variable systemic inflammatory response through endothelial injury, ischemia–reperfusion or hypoperfusion, and immune interactions with bioprosthetic material. This response may be better tolerated in patients with preserved ventricular function but may contribute to clinical decompensation when myocardial reserve is limited (20, 21).

Previous studies have consistently reported associations between metabolic and inflammatory biomarkers and outcomes after TAVR; however, these markers have generally been evaluated as overall risk predictors rather than as context-dependent prognostic signals. In our previous study of 821 patients undergoing TAVR, the triglyceride–glucose (TyG) index was independently associated with mortality across multiple follow-up intervals. Specifically, each 1-unit increase in TyG was associated with a nearly two-fold increase in 30-day mortality (adjusted HR, 1.92), a 62% increase in 1-year mortality (adjusted HR, 1.62), and a 42% increase in 3-year mortality (adjusted HR, 1.42) (14). The present cohort partially, but not completely, overlaps with the institutional TAVR cohort included in our previous study evaluating TyG and mortality after TAVR (14). However, the two studies differ in their study periods, eligibility and exclusion criteria, final sample sizes, analytical populations, exposures, outcomes, and statistical approaches. The previous study included patients treated between 2010 and 2024 and evaluated TyG alone in relation to 30-day, 1-year, and 3-year all-cause mortality. In contrast, the present study was restricted to a more contemporary TAVR era beginning in 2015 and included patients treated through March 2025. This later period was characterized by greater use of newer-generation valve platforms, broader adoption of TAVR, increasingly standardized procedural workflows, and evolving patient-selection and periprocedural practices. In addition, the present study applied different exclusion criteria and used exact matching to construct an LVEF-balanced mortality cohort, as well as a separate time-dependent matched cohort for HVD. Differences in cohort composition, sample size, follow-up, exclusion criteria, and modelling strategy may therefore contribute to differences in effect estimates between the two studies. Most importantly, the previous study did not assess SII, construct the TyG–SII composite index, formally evaluate interaction with baseline LVEF, or examine HVD (14). Accordingly, all analyses involving SII, TSI, the TSI × LVEF interaction, and HVD in the present study are novel. Also, Li et al. demonstrated that each 1-unit increase in TyG was associated with a 441% increase in the hazard of all-cause mortality after TAVR (adjusted HR, 5.41; 95% CI, 4.01–7.32) (22). Similarly, greater inflammatory burden, as assessed by SII, has been associated with adverse outcomes after TAVR. Han et al. reported that each 1-SD increase in SII was associated with an approximately 30% higher risk of major adverse cardiovascular events (11), whereas smaller cohorts demonstrated modest discriminatory performance for mid-term outcomes. Nevertheless, prior studies generally evaluated metabolic and inflammatory markers as additive predictors and did not formally assess whether their prognostic associations varied according to LVEF or ventricular reserve (15). However, most studies did not formally test interactions by LVEF or ventricular reserve, limiting insight into context dependence. Our analysis is, to our knowledge, the first to demonstrate that the prognostic impact of these biomarkers is concentrated in patients with reduced LVEF, rather than being universally predictive regardless of systolic function. By explicitly modeling interaction effects and comparing hazard ratios across strata of LVEF, we extend prior work by providing an effect-modification framework that integrates metabolic dysregulation (TyG), systemic inflammation (SII), and contractile reserve into a unified risk profile.

Importantly, our findings also align with emerging evidence supporting multidomain biomarker strategies in the TAVR setting. Buonpane et al. demonstrated that combining inflammatory and nutritional information through the platelet-to-lymphocyte/albumin ratio was independently associated with one-year all-cause mortality after TAVR and outperformed the prognostic value of single parameters (13). This supports the concept that composite indices may better reflect the complex biological vulnerability of patients undergoing TAVR. Our study extends this concept by evaluating a different biological axis, namely the combined inflammatory–metabolic burden reflected by TyG–SII, and by showing that its prognostic association was not uniform across all patients but appeared concentrated among those with reduced LVEF. Therefore, our results should be interpreted as complementary to prior composite biomarker studies, supporting a more individualized and context-dependent approach to post-TAVR risk stratification.

To our knowledge, this is the first study to demonstrate an independent association between baseline systolic dysfunction and subsequent HVD after TAVR, as contemporary durability research has focused primarily on prosthesis-related and anatomic determinants of HVD rather than ventricular biology. Contemporary registry data have documented the incidence and predictors of HVD using VARC-3 definitions, highlighting the roles of prosthesis factors and hemodynamic metrics, but not baseline LVEF as a predictor of valve deterioration (18). Prior studies on left ventricular ejection fraction in TAVR populations have established that reduced baseline LVEF and lack of post-procedural improvement are associated with worse clinical outcomes and survival, underscoring the biological importance of systolic reserve in long-term follow-up (4, 19, 23). Mechanistically, limited myocardial contractile reserve may lead to persistent adverse transvalvular flow patterns, increased shear stress on prosthetic leaflets, and maladaptive ventricular–valvular interactions, which could accelerate degenerative changes in bioprosthetic valves and contribute to HVD (19).

The observed association between TSI and long-term mortality should also be interpreted in the context of broader clinical vulnerability that may not be fully captured by routinely collected variables. Frailty and impaired nutritional status are established determinants of prognosis among patients undergoing TAVR and may influence survival independently of conventional cardiovascular risk factors (24, 25). Concomitant inflammatory or autoimmune diseases, chronic infections, and recent acute illnesses may alter circulating neutrophil, lymphocyte, and platelet profiles and thereby influence the SII component of TSI independently of the cardiovascular processes under investigation (26, 27). Differences in medical therapy, including anti-inflammatory, lipid-lowering, glucose-lowering, and guideline-directed heart failure therapies, may also influence both biomarker profiles and clinical outcomes (28–30). Consequently, TSI may partly reflect overall biological vulnerability, comorbidity burden, and treatment patterns in addition to the inflammatory–metabolic pathways it was designed to represent. Although matching and multivariable adjustment reduced measured confounding, these findings should be interpreted as demonstrating an association rather than a causal relationship, and the TyG–SII composite should be viewed as an exploratory risk-stratification biomarker requiring prospective validation. In addition, TAVR practice evolved substantially during the study period, including changes in patient selection, device technology, implantation techniques, and peri-procedural management, all of which may have influenced clinical outcomes (31). Although calendar period was incorporated into both the matching strategy and multivariable analyses, residual confounding related to temporal changes in practice cannot be completely excluded. Therefore, despite careful adjustment for measured confounders, these findings should be interpreted as demonstrating an association rather than a causal relationship, and the TyG–SII composite should be viewed as an exploratory risk-stratification biomarker requiring prospective validation.

Although our findings support the prognostic relevance of the TyG–SII composite, the present study was not designed to definitively establish incremental predictive superiority of TSI over TyG or SII alone. Future external validation studies should formally compare individual and composite biomarker models using discrimination, calibration, and reclassification metrics to determine whether TSI provides incremental prognostic value beyond its component indices.

### 4.1 Clinical implications

These findings support a risk-stratification approach that extends beyond LVEF alone by identifying a subgroup of patients with reduced systolic function in whom greater metabolic–inflammatory burden is associated with disproportionately higher risk. Incorporating metabolic and inflammatory indices into pre-TAVR assessment may help identify a high-risk biological phenotype not fully captured by conventional clinical or echocardiographic parameters. These observations also raise the hypothesis that optimization of metabolic and inflammatory status before TAVR may represent a potentially modifiable target, particularly among patients with limited myocardial reserve who may be more vulnerable to periprocedural and postprocedural stress.

### 4.2 Strengths

This study has several notable strengths. First, to our knowledge, this is the first study to report an independent association between baseline left ventricular systolic dysfunction and long-term hemodynamic valve deterioration after TAVR, addressing an important gap in durability research that has traditionally focused on prosthesis- and anatomy-related factors. Second, the study applies an integrative biological framework by jointly evaluating metabolic dysregulation, systemic inflammation, systolic function, and valve durability rather than examining these domains in isolation. Third, the use of matching and multivariable adjustment reduced measured baseline imbalances between patients with reduced and preserved LVEF and enabled a more robust assessment of potential effect modification. Fourth, outcomes were evaluated during long-term follow-up using standardized hemodynamic criteria, thereby enhancing the clinical relevance of the findings. Finally, the identification of a high-risk ventricular–biological phenotype may provide biological insight and support more individualized risk stratification in contemporary TAVR populations.

### 4.3 Limitations

Several limitations should be acknowledged. The retrospective, single-center design limits causal inference and generalizability. Although exact matching and multivariable adjustment were used to reduce measured confounding, the observational design cannot eliminate residual bias from unmeasured or incompletely captured factors. These may include frailty, nutritional status, concomitant inflammatory or autoimmune diseases, chronic infections, recent acute illnesses, differences in treatment intensity, anti-inflammatory medications, lipid-lowering and glucose-lowering therapies, the completeness of guideline-directed medical therapy for heart failure, and procedural evolution over the study period, despite adjustment for the calendar period of TAVR in both the matching strategy and multivariable analyses. Consequently, the observed associations should not be interpreted as causal. Rather, the TyG–SII composite should be considered an exploratory risk-stratification biomarker that requires prospective validation in independent multicenter cohorts before routine clinical implementation. Although multivariable models were intentionally kept parsimonious and constructed according to events-per-variable considerations to reduce the likelihood of overfitting, the relatively limited number of mortality events in the LVEF ≤ 40% subgroup may still have affected the stability and precision of the adjusted estimates. Consequently, these findings should be interpreted with appropriate caution and confirmed in larger prospective cohorts.

Although the standardized TSI × LVEF interaction was statistically significant for long-term all-cause mortality, interaction analyses generally require larger sample sizes than analyses of main effects. Therefore, the precision and reproducibility of the observed effect modification should be confirmed in larger external cohorts. In addition, the number of patients and events within the reduced-LVEF subgroup was relatively limited, which may have affected the stability of the stratum-specific estimates. Furthermore, the analysis of HVD was performed in a smaller time-dependent matched cohort with a limited number of outcome events. Although an association between reduced LVEF and long-term HVD was observed after multivariable adjustment, the borderline statistical significance and limited statistical power reduce the robustness of this finding. In addition, although standardized TSI was significantly associated with HVD within the reduced-LVEF subgroup, the TSI × LVEF interaction was not statistically significant. This subgroup finding should therefore not be interpreted as evidence that the association differed between LVEF strata. Accordingly, the HVD analysis should be considered exploratory and hypothesis-generating until confirmed in larger cohorts with standardized long-term echocardiographic follow-up. Finally, multiple subgroup and interaction analyses may increase the possibility of chance findings; therefore, prospective validation in independent TAVR populations is warranted.

The incremental predictive-performance analysis was restricted to the reduced-LVEF subgroup and was based on a relatively small number of patients and mortality events. Moreover, although the addition of standardized TSI modestly increased Harrell's C-index and improved overall model fit, no formal statistical comparison of the C-index values or external validation was performed. Accordingly, the observed incremental prognostic contribution should be interpreted cautiously and confirmed in independent cohorts.

In addition, metabolic and inflammatory biomarkers were measured at a single time point, precluding assessment of temporal variability or peri-procedural inflammatory dynamics. Furthermore, although TSI was constructed using a multiplicative approach to reflect the concurrent burden of metabolic dysfunction and systemic inflammation, alternative methods of integrating these biomarkers, including additive standardized scores, separate multivariable modeling of TyG and SII, or data-driven weighting approaches, may demonstrate different prognostic performance. These approaches warrant evaluation in future studies to determine the optimal method for integrating metabolic and inflammatory information in patients undergoing TAVR.

## 5 Conclusion

In patients undergoing transcatheter aortic valve replacement, higher inflammatory–metabolic burden, as reflected by the TyG–SII composite index, was independently associated with long-term mortality among those with reduced left ventricular ejection fraction. Formal interaction analysis indicated that baseline LVEF modified the association between standardized TSI and mortality, supporting the potential importance of myocardial vulnerability in determining the prognostic impact of systemic inflammatory–metabolic stress after TAVR. Reduced LVEF was also associated with a higher long-term risk of HVD; however, the corresponding TSI × LVEF interaction was not statistically significant, and this finding should therefore be interpreted cautiously, considered exploratory, and confirmed in larger prospective studies. Combined assessment of ventricular function and inflammatory–metabolic status may improve long-term risk stratification after TAVR, although external validation is required.

## Data Availability

The raw data supporting the conclusions of this article will be made available by the authors, without undue reservation.
