# Supplementary material for: A novel composite inflammatory–metabolic index for long-term prognosis after TAVR: the modifying role of left ventricular ejection fraction
Source: Front Cardiovasc Med. 2026 Aug 25;13:1912257. doi: 10.3389/fcvm.2026.1912257 (PMC13550165; doi:10.3389/fcvm.2026.1912257)
Supplement: Supplementary file 1 [file Datasheet1.docx]

**Supplement 1**

**eTable 1. Multivariable cox regression long-term all-cause mortality among patients with LVEF ≤40%**

**eTable 2. Multivariable cox regression long-term all-cause mortality among patients with LVEF >40%.**

**eTable 3. Multivariable cox regression long-term hemodynamic valve deterioration (HVD) in a 1:4 time-dependent matched cohort (n = 224), matched on sex, age category, calendar period of TAVR, and valve type.**

**eTable 4. Incremental prognostic contribution of standardized TSI for long-term all-cause mortality among patients with LVEF ≤40%**

|  |  |  |  |
| --- | --- | --- | --- |
|  |  |  |  |
|  |  |  |  |
|  |  |  |  |
|  |  |  |  |
|  |  |  |  |

**eTable 1. Multivariable cox regression long-term all-cause mortality among patients with LVEF ≤40%**

| **Variable** | **Reference** | **HR (95% CI)** | **P value** |
| --- | --- | --- | --- |
| Standardized TSI (z-score) | Per 1-SD increase | **1.379 (1.102–1.726)** | **0.005** |
| Age | Per 1-year increase | 1.032 (0.978–1.089) | 0.256 |
| Male sex | Female | 0.841 (0.459–1.542) | 0.577 |
| Atrial fibrillation | No | 1.716 (0.932–3.159) | 0.083 |
| Creatinine | Per 1 mg/dL increase | **1.348 (1.029–1.767)** | **0.030** |

**Hazard ratios were derived from multivariable Cox proportional hazards regression models.
The proportional hazards assumption was not violated.**

**eTable 2. Multivariable cox regression long-term all-cause mortality among patients with LVEF >40%.**

| **Variable** | **Reference** | **HR (95% CI)** | **P value** |
| --- | --- | --- | --- |
| Standardized TSI (z-score) | Per 1-SD increase | 1.023 (0.844–1.239) | 0.818 |
| Age | Per 1-year increase | 0.997 (0.969–1.025) | 0.821 |
| Male sex | Female | **1.556 (1.060–2.285)** | **0.024** |
| BMI | Per 1 kg/m² increase | 0.972 (0.939–1.006) | 0.107 |
| Atrial fibrillation | No | **1.604 (1.113–2.312)** | **0.011** |
| Albumin | Per 1 g/dL increase | **0.416 (0.240–0.722)** | **0.002** |
| Creatinine | Per 1 mg/dL increase | **1.229 (1.022–1.477)** | **0.029** |
| Post-dilatation | No | **1.986 (1.306–3.018)** | **0.001** |
| Annulus area | Per 1 mm² increase | 1.002 (0.999–1.005) | 0.187 |

**Hazard ratios were derived from multivariable Cox proportional hazards regression models. The proportional hazards assumption was not violated.**

**eTable 3. Multivariable cox regression long-term hemodynamic valve deterioration (HVD) in a 1:4 time-dependent matched cohort (n = 224), matched on sex, age category, calendar period of TAVR, and valve type.**

| **Variable** | **Reference** | **HR (95% CI)** | **P value** |
| --- | --- | --- | --- |
| LVEF >40% | Reference | — | — |
| **LVEF ≤40%** | — | **1.710 (1.004–2.912)** | **0.048** |
| Age | Per 1-year increase | 1.036 (0.996–1.077) | 0.079 |
| Gender | Female | 0.862 (0.503–1.475) | 0.587 |
| **Creatinine** | Per 1 mg/dL increase | **1.374 (1.096–1.723)** | **0.006** |
| **Valve size** | Per 1 mm increase | **1.123 (1.029–1.226)** | **0.010** |

**eTable 4. Incremental prognostic contribution of standardized TSI for long-term all-cause mortality among patients with LVEF ≤40%**

| **Model** | **Variables included** | **Harrell’s C-index** | **AIC** | **BIC** | **LR χ² for model comparison** | **P value** |
| --- | --- | --- | --- | --- | --- | --- |
| Base clinical model | Age, sex, atrial fibrillation, and serum creatinine | 0.616 | 343.96 | 356.08 | Reference | — |
| Extended model with TSI | Age, sex, atrial fibrillation, serum creatinine, and standardized TSI | 0.629 | 339.47 | 354.62 | 6.49 | 0.011 |

**Footnotes:**
a Analyses were restricted to patients with baseline LVEF ≤40% (n=153; 51 deaths).
b Harrell’s C-index was used to assess model discrimination.
c The likelihood-ratio test compared the extended model with the nested base clinical model.
d Lower AIC and BIC values indicate improved model fit and parsimony.
e The increase in Harrell’s C-index was descriptive; a formal statistical test comparing the two C-index values was not performed.

**Abbreviations:** AIC, Akaike information criterion; BIC, Bayesian information criterion; CI, confidence interval; LVEF, left ventricular ejection fraction; LR, likelihood ratio; TSI, TyG–SII composite index.
